# How to organize a medical elective

**DOI:** 10.1097/IJ9.0000000000000028

**Published:** 2017-06-15

**Authors:** Georgina Wellstead, Kiron Koshy, Katharine Whitehurst, Buket Gundogan, Alexander J. Fowler

**Affiliations:** a Lister Hospital, Stevenage; b Brighton and Sussex University Hospital; c Royal Devon and Exeter Hospital; d UCL Medical School, University College London; e Guy's and St Thomas' NHS Foundation Trust, London, UK.

**Keywords:** Elective, Medical elective, Organization, Global medicine, Global health, Medical training, Developing world, Developed world

## Abstract

The medical elective is often considered a major highlight of the medical school experience. However, organizing an elective can be a daunting experience. From selecting a destination, to securing bursaries, the road is fraught with uncertainty. In this article, we provide some guidance on how to successfully organize a medical elective. This includes the selection of the destination; options for research or clinical practice and tips for budgeting and acquiring travel bursaries.

## What is an elective?

The medical elective is probably something that has been in the back of your mind since starting university and is often considered a major highlight of the medical school experience. It is a fantastic opportunity to spend around 8 weeks discovering medicine in a different environment, be it within the United Kingdom, or abroad. The elective is also an opportunity for you to distinguish yourself from your fellow students and increase your chances of getting a competitive job.

Choosing and planning the right elective for you can, therefore, be an exciting, yet daunting task. With so many elements to think about, it is difficult to know where to begin!

There are a number of choices to make with regards to electives: what to do and where to go. Some decide on the destination first and then think about what they want to do (specialty and whether mainly clinical or research). Others prefer to focus on the specialty/research interest first. You should make this decision based on what is most important to you and what type of experience you want to get out of your elective.

## Where to go?

An important first step is to decide whether you want to stay in the United Kingdom or travel abroad. Although most people choose to go abroad for their elective, this should not affect your decision. There are plenty of great opportunities within the United Kingdom that would be difficult to take advantage of without taking time out from medical training and work.

If choosing to go abroad, you should consider whether English is spoken, or if you are able to speak the language of the destination, as this could affect your experience in hospital, or your ability to get around. To help with your decision, it may be useful to weigh up the advantages and disadvantages of undertaking an international elective:

Benefits expressed previously by medical students going abroad have included insights into new cultures and health systems, as well as opportunities to challenge themselves academically^1^. Disadvantages, however, have included emotionally challenging situations, difficulties with the distribution of charitable donations, and difficulties identifying and articulating the boundaries of medical practices that the students were comfortable and competent performing^2^.

It is also important to bear in mind that where you choose to go for your elective may help you to decide on your future career path. For example, 1 systematic review suggested that medical students undertaking international medical electives in poorer areas were more inclined to choose a career working with underserved populations and at-risk groups, or in primary care^3^.

Once you have selected a location, make sure to liaise with your medical school early, as there are certain locations that you will not be permitted to go, such as areas of conflict or political unrest. Prevalence of HIV is also an important consideration, as you may be working very hands on in a hospital with poor supervision. The foreign office provides an up-to-date summary of important events/considerations in other countries so make sure you check this^4^.

In addition to this, certain universities will not accept international medical students from universities that do not have a formal partnership, so make sure you check this early to avoid disappointment!

## What to do

Deciding what type of experience you want, and what you would like to get out of your elective is hugely important. Do you want to be very hands-on with a relatively intense timetable, or have a more relaxed experience? Do you want to be in a state-of-the-art hospital in the United States, or work with a poorly funded community outreach team in the developing world?

Most medical schools will require you to fulfil prespecified ‘objectives’ while on your elective; however, these may be very flexible and so can be moulded to your personal preferences in many cases^5^.

You should also consider whether you want to spend your elective getting involved in teaching or research projects. If you have already decided what specialty you would like to go into, tailoring your elective to this can boost your CV and help you down the line when applying for training posts. The experience you have on your elective may also improve your clinical practice. For example, 1 study found that junior doctors who undertook medical electives in palliative care had enhanced competencies in a variety of areas such as patient-centered care, self-awareness, communication skills, professionalism, and psychosocial and spiritual aspects of palliative care^6^.

## Who to go with

Another important consideration is who you want to go with. Do you feel confident enough to go alone, or is it important to you that you go with your friends? Many medical schools allow for a few weeks holiday at the end of the elective period, so this may be a good time to reunite with your friends if you have decided to go on separate electives.

## Budget and funding

It is no secret that electives can end up being very expensive. One obvious benefit of choosing to stay within the United Kingdom is that it will invariably save you some money. If going abroad, however, consider the cost of flights, placement fees, accommodation, insurance, vaccinations, visas, and spending money. It mounts up! You should also include the price of post-exposure prophylaxis, which is vital in areas with high prevalence of HIV, as post-exposure prophylaxis may not be available in these locations. This can cost £120/4-day pack, with not all medical schools providing Discounts^7^.

Travel insurance must be obtained for most electives, which should include health and medicolegal insurance. This may be provided by your current medical insurance organization such as the MDU and MPS, so it is helpful to contact them to see if they will cover your proposed location. If you decide to use a different agency, make sure that you are receiving specialist medical elective travel insurance, as the risks you may be exposed to in hospital are greater than your average traveller. US institutions will also usually require a malpractice policy that insures you $1 million to $3 million. While you are deciding, read the policy carefully before signing up and take down a contact number in case of emergency.

Accommodation is an important issue and is likely to be one of your largest expenses. If you are lucky enough to have relatives in your chosen location, do not be shy, impose! If not, then the elective administrators at your destination can be very helpful in finding cheap, comfortable accommodation, close to your hospital. Bear in mind that you may be working long hours, so being close by is important, especially if the immediate location is not very safe. Many universities also provide accommodation within the elective fee, or at discounted rates, so check with them first.

It is, therefore, vital that before you start planning your elective, you work out what your budget is, as this can have a significant impact on where you are able to go. Your bank may be able to provide you with some advice on this.

Think about what form of currency you will need. Generally a combination of paper currency, traveller’s? and cards are recommended. If you are planning to use your card abroad, let you let your bank know; otherwise you may have your card cancelled or swallowed in a machine! An alternative is to use a travel money card, which allows you to prepay a sum to load onto a card that you can then use abroad without the fees commonly associated with international card usage.

Make sure that you also look into any funding and grants available to you. There are many awards and bursaries out there, so check the websites of the various medical associations and societies, as well as the royal colleges for more details^8^. Some medical schools also offer elective grants and insurance to their students, so it is worth investigating what your university can offer you.

## Planning and organization

Start planning your elective early, as popular destinations will get booked up quickly—sometimes 6 to 18 months in advance (South African Trauma Centres and (HEMS) are booked up years in advance!). If planning to go with a group of friends, you may need to book even earlier to secure your placement. Emailing or calling an elective administrator, or the clinical lead for the department may provide information more efficiently and may also improve your chances of securing a place early in competitive placements.

Your medical school should be able to offer some ideas and contact details based on past electives. However, one of the best things to do is to talk to junior doctors and others who have already been on their elective, to gain contacts and advice on how to go about organizing your own. The Electives Network website, which you can access for free as a member of the Medical Defence Union, is another good source that offers useful information as well as student reviews of their own elective experiences^9^.

The Royal Colleges may also be able to offer advice on electives relevant to particular specialties.

For US electives, electives.us^10^ provides a very useful summary of placement options and is a great place to start.

## Summary

The key things you need to ask yourself when planning your medical elective are:

- Where do you want to go?
- Who do you want to go with?
- What type of experience do you want to have?
- How much will it cost, and what financial support can you access?
- What extras do you need to consider? (visas, vaccinations etc.).
- How far in advance do you need to book?

Finally, and most importantly, make sure that you have a great time!
